# Isogeometric iFEM Analysis of Thin Shell Structures †

**DOI:** 10.3390/s20092685

**Published:** 2020-05-08

**Authors:** Adnan Kefal, Erkan Oterkus

**Affiliations:** 1 Faculty of Engineering and Natural Sciences, Sabanci University, Tuzla, Istanbul 34956, Turkey; adnankefal@sabanciuniv.edu; 2 Integrated Manufacturing Technologies Research and Application Center, Sabanci University, Tuzla, Istanbul 34956, Turkey; 3 Composite Technologies Center of Excellence, Istanbul Technology Development Zone, Sabanci University-Kordsa Global, Pendik, Istanbul 34906, Turkey; 4 PeriDynamics Research Centre, Department of Naval Architecture, Ocean and Marine Engineering, University of Strathclyde, Glasgow G4 0LZ, UK

**Keywords:** inverse finite element method (iFEM), isogeometric analysis, thin and curved shells, shape sensing, structural health monitoring, strain sensors, linear/nonlinear deformation

## Abstract

Shape sensing is one of most crucial components of typical structural health monitoring systems and has become a promising technology for future large-scale engineering structures to achieve significant improvement in their safety, reliability, and affordability. The inverse finite element method (iFEM) is an innovative shape-sensing technique that was introduced to perform three-dimensional displacement reconstruction of structures using in situ surface strain measurements. Moreover, isogeometric analysis (IGA) presents smooth function spaces such as non-uniform rational basis splines (NURBS), to numerically solve a number of engineering problems, and recently received a great deal of attention from both academy and industry. In this study, we propose a novel “isogeometric iFEM approach” for the shape sensing of thin and curved shell structures, through coupling the NURBS-based IGA together with the iFEM methodology. The main aim is to represent exact computational geometry, simplify mesh refinement, use smooth basis/shape functions, and allocate a lower number of strain sensors for shape sensing. For numerical implementation, a rotation-free isogeometric inverse-shell element (isogeometric Kirchhoff–Love inverse-shell element (iKLS)) is developed by utilizing the kinematics of the Kirchhoff–Love shell theory in convected curvilinear coordinates. Therefore, the isogeometric iFEM methodology presented herein minimizes a weighted-least-squares functional that uses membrane and bending section strains, consistent with the classical shell theory. Various validation and demonstration cases are presented, including Scordelis–Lo roof, pinched hemisphere, and partly clamped hyperbolic paraboloid. Finally, the effect of sensor locations, number of sensors, and the discretization of the geometry on solution accuracy is examined and the high accuracy and practical aspects of isogeometric iFEM analysis for linear/nonlinear shape sensing of curved shells are clearly demonstrated.

## 1. Introduction

Structural health monitoring (SHM) is an interdisciplinary procedure that (1) integrates sensing systems into a structure, (2) processes the data collected from the sensing systems in real time, and (3) provides decisive real-time information from the structure about its global and/or local structural state. The main objective of SHM is to detect unusual structural behaviors to pinpoint failures or an unhealthy structural condition. The exercise of SHM serves to increase human and environmental safety, as well as reduce maintenance costs. Therefore, the installation of an SHM system to an engineering structure is essential for the detailed structural management of a structure, including inspection, maintenance, and repair plans [1]. 

A key technology of the SHM process is real-time reconstruction of a structure’s three-dimensional displacement and stress fields, using a network of in situ strain sensors and measured strains, which is commonly referred to as “shape and stress sensing” or “displacement and stress monitoring”. A well-suited algorithm for performing shape and stress sensing of a structure should have the following characteristics. It should be general enough to take complex structural topologies and boundary conditions into account. It has to be robust, stable, and accurate under a wide range of loading conditions, material systems, and inherent errors in the strain measurements. Finally, it should be sufficiently fast for real-time applications [2].

The inverse finite element method (iFEM) is a state-of-the-art methodology originally introduced by Tessler and Spangler [3,4], for the real-time reconstruction of three-dimensional full-field structural displacements, strains, and stresses of structures that are instrumented by strain sensors. The general mathematical concept of the iFEM methodology uses a least-squares variational principle that minimizes the sum of squared errors between the analytical and experimental values of strain measures. It is worth noting that this variational formulation allows the entire structural geometry to be discretized by suitable inverse finite elements (e.g., beam, frame, plate, shell and solid elements), in which measured strains can be adapted to element strains in the least-squares sense.

Despite numerous studies dedicated to the shape sensing of beam and plate structures using analytical approaches [5,6,7] and modal methods [8,9,10], none of these shape-sensing techniques possess the same generalities as what iFEM methodology offers for shape sensing of complex structures. Some of these techniques were recently compared to iFEM methodology for shape sensing of composite wing box [11], where it was demonstrated that the iFEM predicts better and more accurate deformed shapes than Ko theory [6] and modal methods [8]. Moreover, an extensive literature review study of iFEM and other shape-sensing methods can be found in [12]. Herein, we report only those recently published within the context of iFEM. Up until now, three different inverse-plate/shell elements are developed based on Lagrangian shape functions, namely iMIN3 [13], iQS4 [14], and iCS8 [15] elements. Moreover, nonlinear membrane shape and transverse load reconstruction were performed by employing classical shell theory in the iFEM formulation [16]. Besides, various damage detection strategies [17,18] were examined for various engineering structures utilizing the iQS4 element. Additionally, displacement and stress monitoring of complex marine structures [19,20,21] were performed based on iFEM/iQS4 formulation. Furthermore, inverse-beam element formulations were presented for shape sensing of thin/thick beams [2,22]. These inverse elements were numerically and experimentally verified [23,24,25] and sensor placement optimizations were proposed for beam models [26,27]. Lately, various iFEM formulations [28,29,30] were proposed by utilizing kinematic relations of refined zigzag theory [31] for the shape and stress sensing of multilayered composite and sandwich plates/shells. Numerical applications of these iFEM-RZT approaches include the modelling of sensor placement strategy for a tapered plate structure [32]. 

The exact representation of actual structural geometry is crucial for an accurate iFEM analysis of any structure, and especially curved structures. The iFEM analysis of a smoother geometry requires more refined mesh generation for the existing flat inverse-shell elements, e.g., iQS4, iMIN3 elements. A high-fidelity discretization of an iFEM model may require a large number of strain sensors installed on-board structure. Therefore, performing shape sensing and SHM of a complex/curved geometry would be costly using the existing flat inverse-shell elements. Moreover, the shape functions of these flat shell elements are standard polynomial-based functions and limited to only C^0^-continuity for the displacement field. However, a smoother shape sensing can be obtained, if the shape functions ensure a higher continuity $C^{p}\;(p>0)$ throughout the element interior and edge interface. In order to overcome the problems mentioned above and expand the horizon of the iFEM methodology further, the concept of isogeometric analysis (IGA) [33] can be utilized to develop novel isogeometric inverse elements.

Utilizing the non-uniform rational basis splines (NURBS) basis functions, the IGA serves an exact representation of computational geometry, no matter how coarse the discretization. Moreover, it simplifies the mesh refinement by eliminating the need for communication with the computer aided design (CAD) geometry once the initial isogeometric model is constructed. Furthermore, it provides high-order continuity basis functions, and finally knits the mesh generation process within CAD systems. Because of these beneficial aspects, the IGA has received a great deal of attention in the recent years in many different fields of computational mechanics, in particular structural and fluid mechanics. To give an example, Cottrell et al. [34] provided the definitive explanation of the IGA and its future directions. Moreover, IGA has shown advantages over traditional approaches in the context of fluid-structure interaction problems [35], shell and plate problems [36], contact formulations [37], and optimization problems [38].

This study presents a novel “isogeometric iFEM formulation”, which couples the NURBS-based IGA together with the iFEM methodology for the shape sensing of complex/curved thin shell structures. The primary goal is to be geometrically exact, regardless of the discretization size and to obtain a smoother shape sensing, even if using less number of strain sensors. For this purpose, an isogeometric Kirchhoff–Love inverse-shell element (iKLS) is developed, based on the weighted-least-squares functional that uses membrane and bending strain measures consistent with the Kirchhoff–Love shell theory [39,40]. In fact, the Kirchhoff–Love shell model is well suited for thin shell analysis because (1) it disregards both transverse shear deformations and extensibility in thickness direction and (2) the deformation behavior of elastic and homogeneous thin shells is physically dominated by membrane and bending actions. Thus, the Kirchhoff–Love model is more advantageous to use in comparison to the other shell models, because no shear locking occurs if the transverse shear is neglected. The novel iKLS element presented herein employs NURBS basis functions, not only as a geometry discretization technology, but also as a discretization tool for displacement domain. Therefore, this development serves the following beneficial aspects of the IGA for the shape-sensing analysis, based on iFEM methodology: (1) exact representation of computational geometry, (2) simplified mesh refinement, (3) smooth (high-order continuity) basis functions, and finally (4) integration of design and analysis in only one computational geometry. The overall strategy presented in this study is an extended and enhanced version of the authors’ study described in [41]. Particularly, an isogeometric iFEM formulation with more mathematical details on the iKLS implementation is provided and linear/nonlinear displacements of additional problems are estimated by using a low number of strain sensors.

This study is organized as follows: first, Section 2 presents an iFEM formulation for thin and curved shells, which is developed utilizing the kinematics of Kirchhoff–Love shell theory in convected curvilinear coordinates. Besides, a brief summary of B-spline and NURBS basis functions is given and the mathematical structure of the iKLS element, i.e., an example of the isogeometric iFEM formulation, is described. Then, in Section 3, the superior capabilities of iKLS element for shape sensing of curved shells are demonstrated by various case studies including Scordelis–Lo roof, pinched hemisphere, and partly clamped hyperbolic paraboloid. Finally, the conclusions of this study, which indicate the advantages of the iKLS element and isogeometric iFEM methodology, are provided in Section 4.

## 2. Isogeometric iFEM Formulation for Thin Shells

### 2.1. The Inverse Problem: Shape Sensing

**Figure 1:**
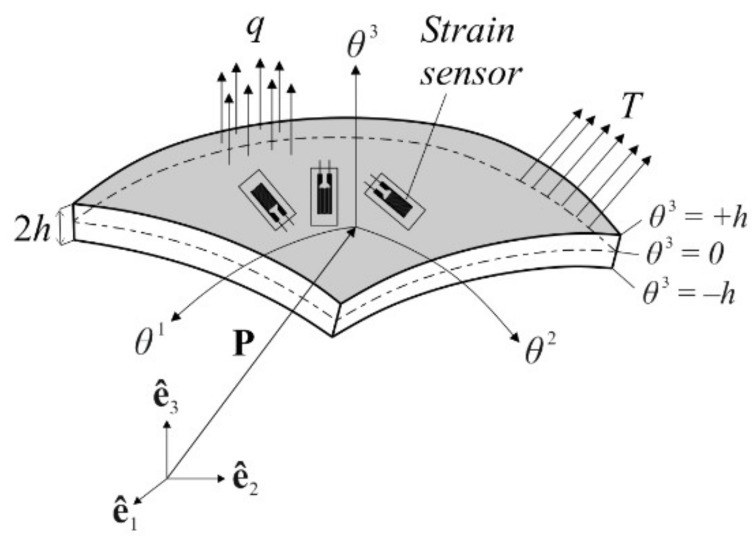
Notation for the curved shell body.

Consider an arbitrary shell body, e.g., a curved shell as depicted in Figure 1, with a uniform thickness 2h that is at least one order of magnitude smaller than the characteristic dimension of the body, such as the span or diameter. To identify a particle (material point) of the curved shell body, we use general convected curvilinear coordinates $\theta^{i}$, for which, unless otherwise specified, Greek indices take the values of 1 and 2, while the Latin indices range from 1 to 3. The coordinate $\theta^{3}\in [-h,+h]$ identifies the thickness direction of the shell and material points located at the mid-surface of the shell are described as $\theta^{3}=0$. Moreover, the in-plane coordinates are represented by $\theta^{\alpha }\in A$ where A denotes the area of the mid-surface. Furthermore, an orientation in three-dimensional Euclidean space, ${\mathbb{R}}^{3}$, is introduced by a fixed orthogonal Cartesian coordinate system. This system has orthonormal basis, ${\hat{e}}_{i}$, pointing the direction of the coordinate axes, as shown in Figure 1. In this regard, linear combination of the ${\hat{e}}_{i}$ vectors and the $P_{i}$ Cartesian coordinates can uniquely establish a position vector Ρ of any arbitrary material point in the shell body as:

(1) $$P\equiv P(\theta^{1},\theta^{2},\theta^{3})=\sum_{i=1}^{3}P_{i}(\theta^{1},\theta^{2},\theta^{3})\;{\hat{e}}_{i}$$

where $P_{i}$ is written as a function of convective coordinates $\theta^{i}$, thus defining the transformations between Cartesian and convective coordinates. 

It is assumed that external forces involving the in-plane and out-of-plane components, T and q, are applied to the shell body. Besides, the rigid body motion of the body is fully constrained. Furthermore, as depicted in Figure 1, strain sensors are attached at discrete locations on the surface of shell, providing real-time strain measurements. The inverse problem at hand is the dynamic tracking of the three-dimensional displacements of the shell body utilizing only the in situ discrete surface strains and boundary restraints. In the following sections, the precise solution of this inverse problem is derived based on an isogeometric iFEM methodology.

### 2.2. Computation of Analytical and Experimental Section Strains for Kirchhoff–Love Shell 

**Figure 2:**
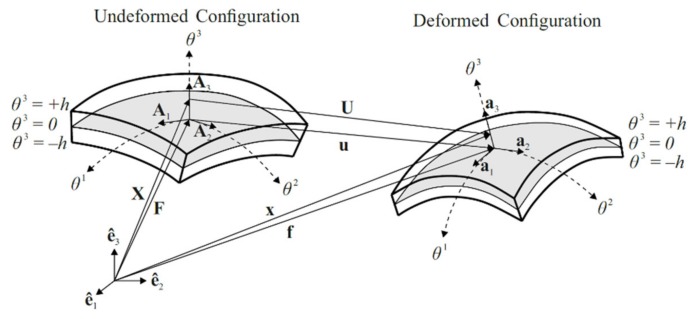
Undeformed and deformed configurations of the shell body.

The arbitrary material points in undeformed (reference) and deformed (current) configurations of the shell body can be described by position vectors X and x, respectively, as shown in Figure 2. The position vector X can be defined by the linear function of thickness coordinate $\theta^{3}$ as:

(2) $$X(\theta^{1},\theta^{2},\theta^{3})=F(\theta^{1},\theta^{2})+\theta^{3}\;A_{3}(\theta^{1},\theta^{2})$$

where F represents a position vector to a material point on the mid-surface in reference configuration and $A_{3}$ denotes a unit-magnitude vector field (the director vector) that is perpendicular to the tangent plane of any point belongs to mid-surface in reference configuration (Figure 2). As given in Equation (2), both F and $A_{3}$ are only functions of the in-plane coordinates $\theta^{\alpha }$. Taking partial derivative of F with respect to $\theta^{\alpha }$ provides the covariant base vectors $A_{\alpha }$ of the mid-surface in reference configuration as:

(3) $$A_{\alpha }=F_{,\alpha }$$

where, hereafter, ${\left(\cdot \right)}_{,\alpha }\equiv \frac{\partial (\cdot )}{\partial \theta^{\alpha }}$ represents a partial derivative with respect to in-plane coordinate $\theta^{\alpha }$. The director vector, $A_{3}$, can be defined by normalized cross product of these covariant base vectors $A_{\alpha }$ as:

(4) $$A_{3}=\frac{A_{1}\times A_{2}}{\left\|A_{1}\times A_{2}\right\|}$$

Analogous to the Equation (2), the position vector x can also be defined by linear functions of thickness coordinate $\theta^{3}$ as:

(5) $$x(\theta^{1},\theta^{2},\theta^{3})=f(\theta^{1},\theta^{2})+\theta^{3}\;a_{3}(\theta^{1},\theta^{2})$$

where f is a position vector to a material point on the mid-surface and $a_{3}$ is the director vector in the current configuration, as shown in Figure 2. According to the 3-parameter Kirchhoff–Love shell model [42], the director vector $a_{3}$ can be defined by the linearized rotation of the director vector $A_{3}$ as:

(6) $$a_{3}=A_{3}+\theta \times A_{3}$$

where θ is the rotation vector and $\theta \times A_{3}$ represents the difference between the directors of the reference and current configurations of the shell body. Accordingly, the displacement vector U of any arbitrary point in the shell body can be defined by subtracting the position vector of undeformed configuration from the position vector of deformed configuration, as:

(7) $$U=x-X=f-F+\theta^{3}\;(a_{3}-A_{3})=u+\theta^{3}\;(\theta \times A_{3})$$

where u is mid-surface displacement vector representing the translational displacements of the mid-surface of shell body from reference to current configuration, as depicted in Figure 2. The orthogonal components of this vector can be defined as a function of in-plane coordinates $\theta^{\alpha }$; that is:

(8) $$u={\left[\begin{array}{ccc} u & v & w \end{array}\right]}^{T}$$

where the functions $u\equiv u(\theta^{1},\theta^{2})$, $v\equiv v(\theta^{1},\theta^{2})$, and $w\equiv w(\theta^{1},\theta^{2})$ represent translations along the covariant Cartesian base vector ${\hat{e}}_{i}$, respectively. According to Kirchhoff–Love theory, the rotation vector θ can be described in terms of in-plane covariant base vectors $A_{\alpha }$ and related rotation angles $\chi_{\alpha }$ as:

(9a) $$\theta =\chi_{1}\;A_{1}+\chi_{2}\;A_{2}$$

with

(9b) $$\chi_{1}=\frac{(a_{2}-A_{2})\cdot A_{3}}{\left\|A_{1}\times A_{2}\right\|}=\frac{u_{,2}\cdot A_{3}}{\left\|A_{1}\times A_{2}\right\|}$$

(9c) $$\chi_{2}=-\frac{(a_{1}-A_{1})\cdot A_{3}}{\left\|A_{1}\times A_{2}\right\|}=-\frac{u_{,1}\cdot A_{3}}{\left\|A_{1}\times A_{2}\right\|}$$

where $u_{,\alpha }$ denoting the partial derivatives of u with respect to $\theta^{\alpha }$ are utilized to define rotation angles $\chi_{\alpha }$. Therefore, the rotation vector θ is a function of $u_{,\alpha }$, so that the orthogonal components of u, namely (u,v,w), are the only unknowns, i.e., kinematic variables, to predict the displacement vector U in the analysis. 

The partial derivatives of the displacement field U, with respect to curvilinear convective coordinates $\theta^{i}$, can be evaluated as:

(10a) $$U_{,\alpha }=u_{,\alpha }+\theta^{3}(\theta_{,\alpha }\times A_{3}+\theta \times A_{3,\alpha })$$

(10b) $$U_{,3}=\theta \times A_{3}$$

where, hereafter, ${\left(\cdot \right)}_{,3}\equiv \frac{\partial (\cdot )}{\partial \theta^{3}}$ represents a partial derivative with respect to thickness coordinate $\theta^{3}$. Moreover, the covariant base vectors $g_{i}$ of the shell body can be calculated as:

(11a) $$g_{\alpha }=X_{,\alpha }=A_{\alpha }+\theta^{3}A_{3,\alpha }$$

(11b) $$g_{3}=X_{,3}=A_{3}$$

Using Equations (10a) and (11a), the linearized Green–Lagrange strain tensor defined in convected curvilinear coordinates gives rise to in-plane strains:

(12) $$\left\{\begin{array}{c} \varepsilon_{11}\\ \varepsilon_{22}\\ \gamma_{12} \end{array}\right\}=\left\{\begin{array}{c} U_{,1}\cdot g_{1}\\ U_{,2}\cdot g_{2}\\ U_{,1}\cdot g_{2}+U_{,2}\cdot g_{1} \end{array}\right\}=\left\{\begin{array}{c} e_{1}\\ e_{2}\\ e_{3} \end{array}\right\}+\theta^{3}\left\{\begin{array}{c} \kappa_{4}\\ \kappa_{5}\\ \kappa_{6} \end{array}\right\}\equiv e(u)+\theta^{3}\kappa (u)$$

where the vectors e(u) and κ(u) represent membrane strain measures and bending curvatures, respectively, and their components can be explicitly expressed as:

(13a) $$e_{1}=u_{,1}\cdot A_{1}$$

(13b) $$e_{2}=u_{,2}\cdot A_{2}$$

(13c) $$e_{3}=u_{,1}\cdot A_{2}+u_{,2}\cdot A_{1}$$

(13d) $$\kappa_{4}=u_{,1}\cdot A_{3,1}+(A_{3}\times A_{1})\cdot \theta_{,1}+\underset{=0}{\underset{︸}{(\theta \times A_{3,1})\cdot A_{1}}}$$

(13e) $$\kappa_{5}=u_{,2}\cdot A_{3,2}+(A_{3}\times A_{2})\cdot \theta_{,2}+\underset{=0}{\underset{︸}{(\theta \times A_{3,2})\cdot A_{2}}}$$

(13f) $$\begin{array}{lll} \kappa_{6}=u_{,1}\cdot A_{3,2}+u_{,2}\cdot A_{3,1} & +(A_{3}\times A_{2})\cdot \theta_{,1} & +(A_{3}\times A_{1})\cdot \theta_{,2}\\ & & +\underset{=0}{\underset{︸}{(\theta \times A_{3,1})\cdot A_{2}}}+\underset{=0}{\underset{︸}{(\theta \times A_{3,2})\cdot A_{1}}} \end{array}$$

where all strain contributions of $(\theta \times A_{3,\alpha })\cdot A_{\beta }$ vanish identically, because the vectorial quantities obtained from the cross products of θ and $A_{3,\alpha }$ are normal to the mid-surface of the shell body so that the scalar multiplication of these vectorial quantities and $A_{\beta }$ provides the final results as $(\theta \times A_{3,\alpha })\cdot A_{\beta }=0$. In addition to the in-plane strains, the linearized Green–Lagrange strain tensor defines the transverse-shear strains utilizing Equations (10b) and (11b) as:

(14) $$\begin{array}{ll} \gamma_{\alpha 3}=U_{,\alpha }\cdot g_{3}+U_{,3}\cdot g_{\alpha } & =\underset{=0}{\underset{︸}{u_{,\alpha }\cdot A_{3}+(\theta \times A_{3})\cdot A_{\alpha }}}\\ & +\theta^{3}\;[\underset{=0}{\underset{︸}{(\theta_{,\alpha }\times A_{3})\cdot A_{3}}}+\underset{=0}{\underset{︸}{(\theta \times A_{3,\alpha })\cdot A_{3}+(\theta \times A_{3})\cdot A_{3,\alpha }}}]=0 \end{array}$$

Thus, the Kirchhoff–Love shell model exhibits zero transverse-shear strains, $\gamma_{\alpha 3}=0$. This indicates that the deformations of the shell body will be physically dictated by only membrane and bending actions.

**Figure 3:**
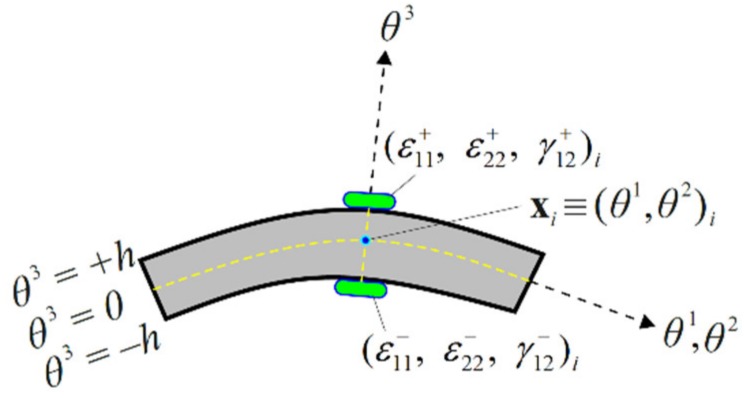
Discrete surface strains measured at $\left(x_{i},\pm h)\;\;(i=1,2,\ldots ,n_{s}\right)$.

To compute the experimental section strains, the strain rosettes are located on the top and bottom surface of the curved shell as depicted in Figure 3. Using these surface strain measurements, the in situ membrane strain measures and bending curvatures, $E_{i}$ and $K_{i}$, that correspond to their analytic counterparts e(u) and κ(u), given by Equations (13a)–(13f), can be determined at the location $x_{i}={\left(\theta^{1},\theta^{2}\right)}_{i}$ on the mid-surface of the shell, as follows:

(15a) $${Ε}_{i}=\frac{1}{2}{\left\{\begin{array}{c} \varepsilon_{11}^{+}+\varepsilon_{11}^{-}\\ \varepsilon_{22}^{+}+\varepsilon_{22}^{-}\\ \gamma_{12}^{+}+\gamma_{12}^{-} \end{array}\right\}}_{i}\;\;(i=1,2,\ldots ,n_{s})$$

(15b) $${Κ}_{i}=\frac{1}{2h}{\left\{\begin{array}{c} \varepsilon_{11}^{+}-\varepsilon_{11}^{-}\\ \varepsilon_{22}^{+}-\varepsilon_{22}^{-}\\ \gamma_{12}^{+}-\gamma_{12}^{-} \end{array}\right\}}_{i}\;\;(i=1,2,\ldots ,n_{s})$$

where ${\left(\varepsilon_{11}^{+},\;\varepsilon_{22}^{+},\;\gamma_{12}^{+}\right)}_{i}$ and ${\left(\varepsilon_{11}^{-},\;\varepsilon_{22}^{-},\;\gamma_{12}^{-}\right)}_{i}$ are the surface strains measured at $n_{s}$ discrete locations $\left(x_{i},\pm h)\;\;(i=1,2,\ldots ,n_{s}\right)$ with the superscripts ‘+’ and ‘–’ denoting the quantities that correspond to the top and bottom surface locations, respectively.

Applying curve-fitting or smoothing techniques [43] to raw strain data or discrete section strains, the continuous forms of experimental sections can be obtained as E and K, where i subscript is removed to signify spatial continuity, i.e., defined everywhere in the shell domain.

### 2.3. The Weighted-Least-Squares Functional and Its Discretization Using NURBS Basis Functions 

According to the iFEM methodology, a weighted-least-squares functional, Φ(u), that takes into account the membrane and bending deformations of the current Kirchhoff–Love shell model, can be established as:

(16) $$\Phi (u)=\frac{1}{A}\int_{A}\left(w_{e}{\left\|e(u)-Ε\right\|}^{2}+w_{\kappa }{\left(2h\right)}^{2}{\left\|\kappa (u)-Κ\right\|}^{2}\right)dA$$

where $w_{e}$ and $w_{\kappa }$ are positive valued weighting constants of the membrane strain measures and bending curvatures, respectively. In case of full sensor model, they can be set to unity, whereas they are set to a small number compared to unity for positions/elements with no sensor. In Equation (16), the continuous experimental section strains, E and K, are used for the notational brevity only. In fact, they are not necessarily need to be available in the iFEM analysis, since one can directly use the available discrete data, $E_{i}$ and $K_{i}$, when performing the area-integrals in Equation (16). This part will be detailed in the remainder of this section. Overall, the present isogeometric iFEM approach does not enforce ‘a priori’ smoothing of the surface measurements and allows the direct usage of discrete strain data. The Euclidian norms given in Equation (16) can be expressed as dot products of vectors, i.e., ${\left\|\varphi \right\|}^{2}\equiv \varphi^{T}\varphi$, then, the least-squares functional can be written more explicitly, as:

(17) $$\Phi (u)=\frac{1}{A}\int_{A}\left(w_{e}e^{T}e+w_{\kappa }{\left(2h\right)}^{2}\kappa^{T}\kappa -2\left(w_{e}e^{T}Ε+w_{\kappa }{\left(2h\right)}^{2}\kappa^{T}Κ\right)+w_{e}{Ε}^{T}Ε+w_{\kappa }{\left(2h\right)}^{2}{Κ}^{T}Κ\right)dA$$

The utility of weighting coefficients for sparse sensor placement models were clearly discussed in various iFEM studies [15,21,29]. For instance, if an experimentally measured strain component is not available in any case, the Equation (17) can be reduced to squared norms of only analytical section strains as:

(18) $$\Phi (u)=\frac{\lambda }{A}\int_{A}\left(e^{T}e+{\left(2h\right)}^{2}\kappa^{T}\kappa \right)dA\;\mathrm{for}\;(w_{e}=w_{\kappa }=\lambda )$$

where the corresponding weighting coefficient is set to be small, e.g., $\lambda =10^{-5}$. More details on the weighting strategies can be found in [15].

Since a large group of literature have already focused on the NURBS basis functions [44,45], only a very brief summary is provided here to establish the notation used in the rest of the study. Three independent parameters ξ, η, and ζ that unify a parameter space (ξ,  η,  ζ) are utilized to describe the B-spline and NURBS basis functions. A B-spline curve can be constructed using a knot vector in one dimension and a vector of control points. A knot vector contains a non-decreasing set of coordinates in the parameter space. For example, a knot vector in one dimension can be defined as $\Xi =\{\xi_{1},\xi_{2},\ldots ,\xi_{n+p+1}\}$ where $\xi_{i}\in \xi \in \mathbb{R}$ is the $i^{th}$ knot, i is the knot index, n is the number of basis functions, and p is the polynomial order (degree). If a knot vector whose first and last knots have multiplicity p+1 for a B-spline of polynomial degree p, this knot vector is called an open knot vector. Each repetition of any knot in the interior of a knot vector locally decreases the degree of continuity by one. The boundaries of the elements in the parametric space are defined based on the locations of the knots. 

According to the Cox–De Boor recursion formula [46,47], the set of B-spline basis functions can be defined through a recursive relation, starting with piecewise constants (p=0):

(19a) $$N_{i}^{0}(\xi )=\left\{\begin{array}{ll} 1 & \mathrm{if}\;\xi_{i}\leq \xi <\xi_{i+1}\\ 0 & \mathrm{otherwise} \end{array}\right.$$

For p=1,2,3,…, they are defined by:

(19b) $$N_{i}^{p}(\xi )=\frac{\xi -\xi_{i}}{\xi_{i+p}-\xi_{i}}N_{i}^{p-1}(\xi )+\frac{\xi_{i+p+1}-\xi }{\xi_{i+p+1}-\xi_{i+1}}N_{i+1}^{p-1}(\xi )\;$$

where the fractions of 0/0 are defined as zero. The B-spline basis functions are generally not interpolatory, except at the boundaries. In addition, they satisfy the partition of unity condition. Moreover, they are positively valued everywhere and a basis function of degree p can span up to p+1 elements. The generalized mathematical form of the B-splines, i.e., NURBS basis functions, can be constructed through projective transformation of B-splines, by utilizing geometric weights defined at each control point. The NURBS curves and surfaces have the same properties as B-spline curves and surfaces. In three-dimensional space, a shell surface, S(ξ,η), can be readily defined by associating the control net, $s_{ij}$, with the two dimensional NURBS basis functions, $R_{ij}^{pq}(\xi ,\eta )$, as:

(20a) $$S(\xi ,\eta )=\sum_{i=1}^{n}\sum_{j=1}^{m}R_{ij}^{pq}(\xi ,\eta )\;s_{ij}$$

(20b) $$R_{ij}^{pq}(\xi ,\eta )=\frac{N_{i}^{p}(\xi )\;M_{j}^{q}(\eta )\;{\tilde{w}}_{ij}}{\sum_{k=1}^{n}\sum_{l=1}^{m}N_{k}^{p}(\xi )\;M_{l}^{q}(\eta )\;{\tilde{w}}_{kl}}$$

where ${\tilde{w}}_{ij}$ is positive-valued constant and referred to as weight of $ij^{th}$ control point. Note that except the control points on the both ends of the surface, the control points are not necessarily located on the surface that they define. The pq superscript denoting the order of the individual B-splines can be removed for brevity of the notation. In addition, for simplicity, the subscript ij, henceforward, is replaced by a single subscript i. Therefore, Equation (20a) can concisely be rewritten as:

(21) $$S(\xi )=\sum_{i=1}^{N_{cp}}R_{i}(\xi )\;s_{i}\equiv \sum_{i}R_{i}\;s_{i}$$

where $\xi \equiv \left(\xi ,\eta \right)$ represents the two-dimensional parameter space and $N_{cp}=n\times m$ denotes the number of control points, and hereafter, they will be omitted for conciseness of the summations including basis functions. 

**Figure 4:**
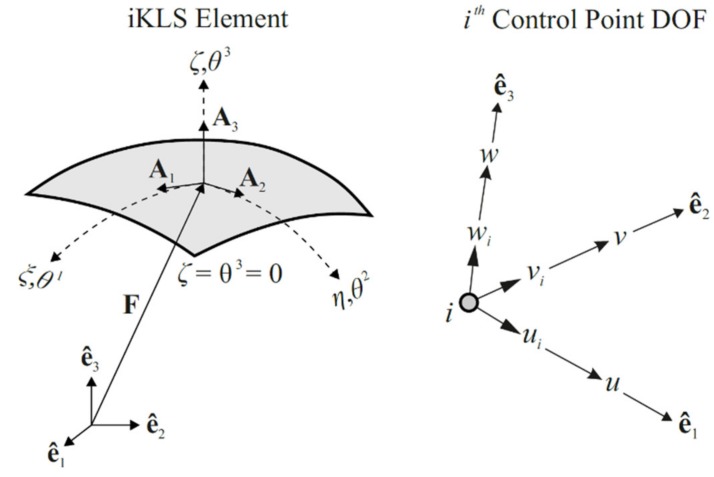
The isogeometric Kirchhoff–Love inverse-shell (iKLS) element and displacement DOF of $i^{th}$ control point.

An isogeometric Kirchhoff–Love inverse-shell element, named “iKLS”, is developed to discretize the iFEM weighted-least-squares formulation. This development couples the NURBS-based IGA together with the iFEM methodology for shape-sensing analysis, thus leads a novel “isogeometric iFEM formulation”. In the following derivations, the parametric coordinates (ξ, η, ζ) are associated with general convected curvilinear coordinates $\theta^{i}$. Hence, the coordinates $\xi \equiv (\xi ,\eta )\equiv (\theta^{1},\theta^{2})$ represent the in-plane coordinates and the coordinate $\zeta =\theta^{3}$ indicates the thickness direction of the iKLS element. The position vectors $A_{i}$ and F, the displacement degrees-of-freedom (DOF) $\left(u_{i},\;v_{i},\;w_{i}\right)$ of $i^{th}$ control point, and the kinematic variables (u, v, w) of the iKLS formulation are illustrated in Figure 4.

To develop the iKLS element formulation, first, the position vector F to a material point on the mid-surface, which is used to define the Equation (2), can be described by the finite sum of two-dimensional NURBS basis functions, as:

(22) $$F=\sum_{i=1}^{n_{cp}}R_{i}^{e}(\xi )\;P_{i}^{e}\equiv \sum_{i}R_{i}P_{i}\;\;\;\;\;(e=1,2,\ldots n_{el})$$

where $n_{el}=(n-p)\times (m-q)$ is the total number of elements available on S surface, $R_{i}^{e}(\xi )\equiv R_{i}$ represents the NURBS basis functions belonging to an individual element, $P_{i}^{e}\equiv P_{i}$ is the coordinates of the control points that defines the physical geometry of an iKLS element with $i=1,2,\ldots ,n_{cp}$ index being the local identities of the control points, and $n_{cp}=(p+1)\times (q+1)$ is the total number of control points of the element. Secondly, taking the partial derivatives of F with respect to parametric coordinates ξ and η, the covariant base vectors $A_{\alpha }$ of the mid-surface given by Equation (3) can be obtained as:

(23a) $$A_{\alpha }=F_{,\alpha }=\sum_{i}R_{i,\alpha }P_{i}$$

where the first-order derivatives of the NURBS shape function are denoted as:

(23b) $$R_{i,1}=R_{i,\xi },\;\;\;R_{i,2}=R_{i,\eta }$$

The (u, v, w) kinematic variables, i.e., the orthogonal components of the mid-surface displacement vector u given by Equation (8), can be interpolated using translation DOF $\left(u_{i},\;v_{i},\;w_{i}\right)$ of control points and the same NURBS basis functions $R_{i}(\xi )$ used for the physical geometry discretization. These interpolations are explicitly given as:

(24a) $$u=\sum_{i}R_{i}u_{i}^{e}$$

(24b) $$u_{i}^{e}={\left[\begin{array}{ccc} u_{i} & v_{i} & w_{i} \end{array}\right]}^{T}\;\;\;(i=1,2,\ldots ,n_{cp})$$

Substituting Equations (23a), (23b) and (24a), (24b) into Equations (13a)–(13f), the membrane strain measures and bending curvatures can be expressed in terms of the displacement vector $u^{e}$ of an iKLS element, as:

(25a) $$\left[\begin{array}{cc} e & \kappa \end{array}\right]=\left[\begin{array}{cc} B_{m}u^{e} & B_{b}u^{e} \end{array}\right]$$

with

(25b) $$u^{e}={\left[\begin{array}{cccc} u_{1}^{e} & u_{2}^{e} & \cdots & u_{n_{cp}}^{e} \end{array}\right]}^{T}$$

where the displacement vector $u^{e}$ contains all translational DOFs of the control points belonging to an iKLS element, and the matrices $B_{\alpha }\equiv B_{\alpha }(\xi )\;(\alpha =m,b)$ are functions of parametric coordinates and contain the derivatives of the NURBS basis functions. These matrices establish the strain-displacement relations of the element and are explicitly given in Appendix A.

Substituting the analytical section strains given by Equations (25a), (25b) into Equation (17), the weighted-least-squares functional, $\Phi (u)\approx \Phi (u^{e})$, can be approximated for an individual iKLS element, accounting for its membrane and bending deformations. All strain compatibility relations are explicitly satisfied based on this approximation/discretization; therefore, the $\Phi (u^{e})$ functional can be minimized with respect to displacement vector $u^{e}$ of an iKLS element, as:

(26) $$\frac{\partial \;\Phi (u^{e})}{\partial \;u^{e}}=2\;(\Gamma^{e}u^{e}-\varepsilon^{e})=0\Rightarrow \Gamma^{e}\;u^{e}=\varepsilon^{e}$$

where $\Gamma^{e}$ is the element left-hand-side matrix, which are independent from experimental strain measures, thus, it needs to be constructed once during the real-time shape-sensing analysis. On the other hand, the element right-hand-side vector, $\varepsilon^{e}$, given in Equation (26), is a function of the measured strain values and needs to be updated for each strain-data acquisition in real time. The $\Gamma^{e}$ matrix can be explicitly written in terms of the $B_{\alpha }\;(\alpha =m,b)$ matrices and their corresponding weighting constants $w_{\alpha }\;(\alpha =e,\kappa )$ as:

(27) $$\Gamma^{e}=\frac{1}{A}\int_{A}\left(w_{e}B_{m}{}^{T}B_{m}+w_{\kappa }{\left(2h\right)}^{2}B_{b}{}^{T}B_{b}\right)dA$$

Besides, the $\varepsilon^{e}$ vector is a function of experimental section strains, and is given by:

(28) $$\varepsilon^{e}=\frac{1}{A}\int_{A}\left(w_{e}B_{m}{}^{T}Ε+w_{\kappa }{\left(2h\right)}^{2}B_{b}{}^{T}Κ\right)dA$$

The integrations given in Equations (27) and (28) can be suitably calculated through the Gauss quadrature method, for which the area-integral of a function is defined as weighted sum of the function values evaluated at predefined Gauss points. Since the $B_{\alpha }\;(\alpha =m,b)$ matrices are continuous in the element domain, all Gauss points required for full integration over the mid-surface of the iKLS element can be directly used when performing the area integration in Equation (27). On the contrary, the experimental section strains, Ε and Κ, may not be available in the continuous form, as given in Equation (28). In this case, the reduced-integration can be performed for Equation (28) with fewer integration points, where the discrete values of experimental strain measures, ${Ε}_{i}$ and ${Κ}_{i}$, should be available as well. As detailed in [15], the one-point Gauss integration can be practically chosen to calculate Equation (28), requiring only a single set of experimental strain measures collected at the centroid of the iKLS element. Alternatively, the area integration in Equation (28) can be performed by employing full-integration points and assuming that the ${Ε}_{i}$ and ${Κ}_{i}$ values at the Gauss points have an identical average-value in the element domain. Note that, finally, if such discrete experimental values are somehow missing, then small weighting coefficients can be assigned to their associated analytical counterparts when calculating Equation (27).

Using the element matrix equations, global linear equation system of a given isogeometric discretization (i.e., composed of $n_{el}$ number of elements) can be obtained as:

(29a) $$\bar{\Gamma }\bar{U}=\bar{\varepsilon }$$

with

(29b) $$\bar{\Gamma }=\overset{n_{el}}{\underset{e=1}{\cup }}\Gamma^{e},\;\bar{U}=\overset{n_{el}}{\underset{e=1}{\cup }}u^{e},\;\bar{\varepsilon }=\overset{n_{el}}{\underset{e=1}{\cup }}\varepsilon^{e}$$

where the symbol $\overset{n_{el}}{\underset{e=1}{\cup }}$ signifies the assembly process of classical finite element or isogeometric analysis. Moreover, the $\bar{\Gamma }$ matrix is the global shape matrix of the discretization, and $\bar{U}$ vector is the global displacement DOF of the whole structure, and $\bar{\varepsilon }$ is the global vector of the experimentally measured strains. Here, we are interested in finding the displacements of the discretization. For this purpose, problem-specific constraint boundary conditions can be imposed to the Equation (29a) and the reduced set of matrix-vector system can be obtained as:

(30) $${\bar{\Gamma }}_{R}{\bar{U}}_{R}={\bar{\varepsilon }}_{R}\;{\bar{U}}_{R}={\bar{\Gamma }}_{R}{}^{-1}{\bar{\varepsilon }}_{R}$$

where ${\bar{\Gamma }}_{R}$ matrix becomes the well-posed (i.e., positive definite) matrix and can be readily inverted herein for the solution of the unknown $\;{\bar{U}}_{R}$ displacements. Finally, combining these reconstructed displacements with the known displacement boundary conditions, the $\bar{U}$ vector can be obtained for each strain-data acquisition in real time, hence providing the deformed shape of the shell surface. Overall, the present isogeometric iFEM formulation attempts the solution of small/linear displacements without rigid-body motions. However, in case of nonlinear and large deformation reconstruction, as long as the experimental surface strains contain the nonlinear effects, the present formulation can be suitably utilized in an incremental sense of small strain/deformations, thereby enabling one to perform the incremental nonlinear shape-sensing analysis of the shell structures undergoing large deformations. 

## 3. Numerical Examples

In the following section, the shape-sensing capability of the iKLS element is assessed and validated, solving three different shell problems. First, Scordelis–Lo roof and the pinched hemisphere problems are solved as benchmark problems for validating the membrane and bending capability of the iKLS element, respectively. In fact, these problems are the first two test cases of a very well-known shell obstacle course proposed and studied earlier [48,49]. Moreover, hyperbolic paraboloid [50] has been widely used in the literature for evaluating the shell elements’ performance, because the shell structure is subjected to stress states of varying complexity. Therefore, after validating the membrane and bending capability of the iKLS element, the partly clamped hyperbolic paraboloid problem is solved to better assess the ability of the iKLS element against the locking phenomenon. 

### 3.1. Scordelis–Lo Roof

A portion of a cylindrical shell whose end sections are fixed by rigid diaphragms has a radius of *r* = 25 m, length of *L* = 50 m, and thickness of 2*h* = 0.25 m. The constraint boundary conditions pertaining to rigid diaphragms can be specified as V=W=0. The cylindrical shell made of an isotropic material having an elastic modulus of *E* = 432 MPa, a zero Poisson’s ratio *v* = 0, and a density of *ρ* = 4 kg/m^3^. A distributed loading represented as a gravitational load *g* = 90 m/s^2^ is applied in negative *Z* direction. This problem was originally solved in [51], and then it has been extensively studied by many researchers (e.g., [48]) and is the so-called Scordelis–Lo roof. 

**Figure 5:**
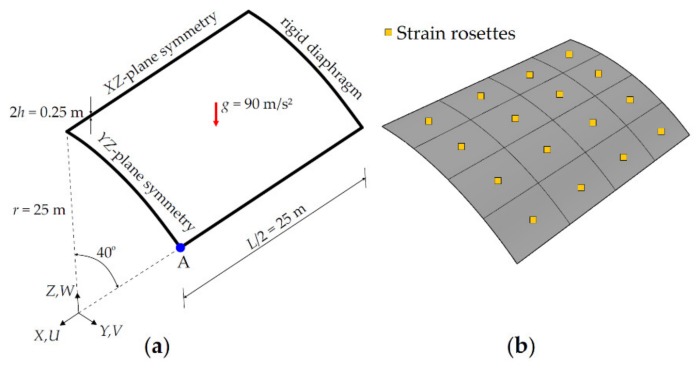
(**a**) Scordelis–Lo roof with symmetric boundary conditions; (**b**) its discretization (n^e^ = 4), using iKLS elements with top- and bottom-surface strain rosettes per each element.

In this section, the Scordelis–Lo roof is analyzed once again using the isogeometric iFEM methodology to validate membrane capability of the iKLS element, because a substantial part of the strain energy is exhibited by membrane strain energy during the deformation of the roof. There is no need to model the whole roof, because the applied boundary conditions and geometry of the roof are suitable for taking advantage of the symmetry conditions. Therefore, the following iFEM and direct FEM models are defined over a quarter of the geometry, and the relevant symmetry constraint boundary conditions are applied, as shown in Figure 5a.

To establish an accurate reference solution, a convergence study was performed using direct FEM analyses, utilizing an in-house FEM code. The most refined mesh consisted of 8100 uniformly distributed rectangular elements, possessing 49686 DOF. The vertical displacement along the *Z*-direction at the midpoint of the lateral side (i.e., point A as depicted in Figure 5a is denoted by the symbol $W_{A}$. As a result of high-fidelity FEM analysis performed, the value of this vertical displacement is obtained as $W_{A}=-0.3017\;m$, which agrees very well with the reference solution predicted in [48] as $W_{A}=-0.3024\;m$. Thus, the FEM deflections and rotations can be safely used to compute the simulated strain-sensor strains in the following iFEM analysis.

In the present iFEM analysis, the Scordelis–Lo roof is analyzed using seven different iKLS discretization, where the edges of the roof are divided by the same number of element subdivisions (n^e^ = 2,…,8). For each iKLS model, the polynomial degrees of the NURBS shape functions are fixed to p=q=8 and C^1^-continuity is attained across an interior element boundary. Every single iKLS element is instrumented with two strain rosettes, one on the centroid of the top surface and the other one on the centroid of the bottom surface. In Figure 5b, an example of strain rosette configurations is shown for iKLS discretization (n^e^ = 4). In the rest of the study, the area-integration in Equation (28) is calculated over the iKLS element domain, by using full (gauss) integration points and assuming that all gauss points possess the same values of experimental section strains obtained from a single set of experimental surface rosettes available in the element domain.

**Figure 6:**
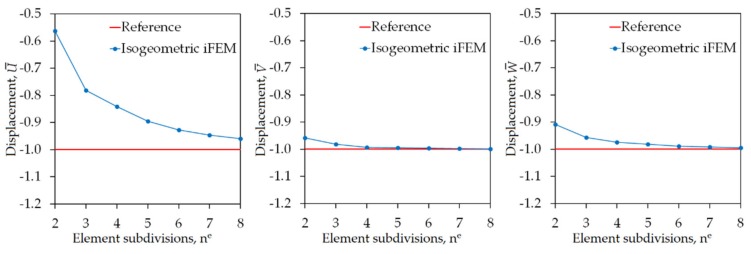
Convergence of maximum values of $\bar{U}$,$\bar{V}$,$\bar{W}$ displacements versus increasing number of element subdivisions n^e^ for Scordelis–Lo roof.

To assess the accuracy of the displacement predictions, it would be convenient to use maximum values of displacements obtained from the high-fidelity FEM solutions (reference) as normalization factors. These normalizations are given as:

(31) $$\bar{\chi }=\chi \;/\;\left|\chi_{\max}^{\mathrm{FEM}}\right|\;\;\;\;(\chi =U,V,W)$$

where maximum values of the reference displacements are $U_{\max}^{\mathrm{FEM}}=-0.0125\;m$, $V_{\max}^{\mathrm{FEM}}=-0.1588\;m$, and $W_{\max}^{\mathrm{FEM}}=-0.3017\;m$. The legend “iFEM” represents the isogeometric iFEM solutions, whereas the legend “Reference” represents the high-fidelity FEM solutions (henceforward, refer to all graphs). In Figure 6, maximum values of the iFEM and FEM predictions for the $\bar{U}$, $\bar{V}$, $\bar{W}$ normalized displacements given in Equation (31) are plotted, versus the number of element subdivisions (n^e^) of the Scordelis–Lo roof, respectively.

These graphs show that the iFEM predictions for the $\bar{V}$ and $\bar{W}$ displacements convergence to the reference solution much quicker than the iFEM predictions for the $\bar{U}$ displacement. In fact, the deformed shape (total deformation) of the roof is mainly caused by the maximum reference displacement $V_{\max}^{\mathrm{FEM}}$ and $W_{\max}^{\mathrm{FEM}}$, because these reference displacements are at least ten times greater than the displacement $U_{\max}^{\mathrm{FEM}}$; hence, the convergence of iFEM predictions for displacement $\bar{U}$ will not play a distinguished role for the real-time reconstruction of the total deformation. As a result, the results depicted in Figure 6 confirm that the isogeometric iFEM formulation of the iKLS element predicts displacements that are as accurate as those of the reference solutions.

**Figure 7:**
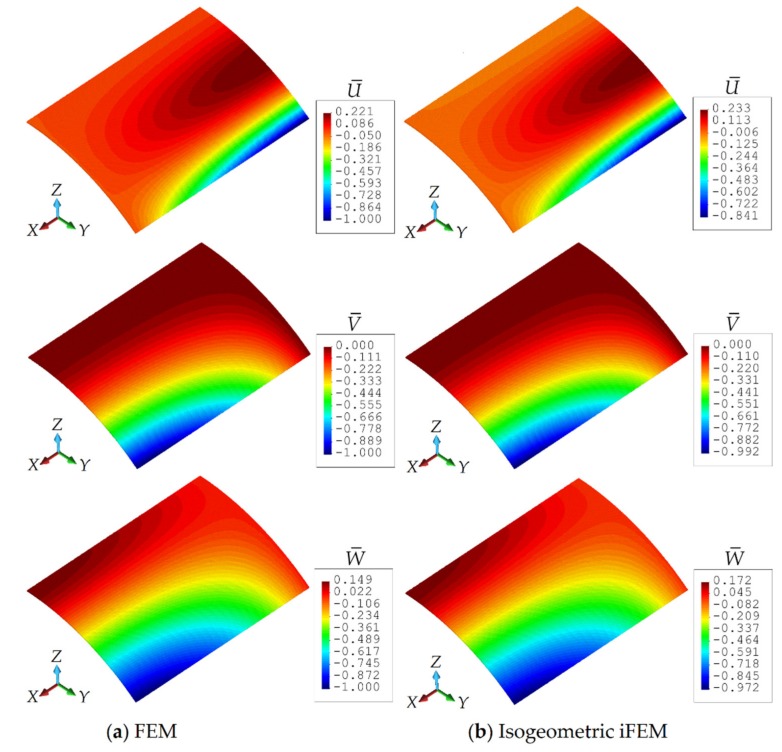
Contour plots of $\bar{U}$,$\bar{V}$,$\bar{W}$ displacements for Scordelis–Lo roof: Comparison between (**a**) high-fidelity finite element method (FEM) and (**b**) isogeometric inverse finite element method (iFEM) (n^e^ = 4) analyses.

Moreover, in Figure 7, the iFEM and FEM contour plots for $\bar{U}$, $\bar{V}$, and $\bar{W}$ are presented, showing the results that are graphically indistinguishable. In these contour plots, the displacement results pertaining to iFEM analysis are obtained using the iKLS discretization (n^e^ = 4), with 16×2 strain rosettes in total. The percent difference between the iFEM and FEM solutions for the maximum values of $\bar{U}$, $\bar{V}$, and $\bar{W}$ are respectively 15.9%, 0.8%, and 2.8%. Even though the percentage difference for displacement $\bar{U}$ is relatively high, as explained in the above paragraph, this displacement does not contribute much to the deformed shape. Therefore, these percent differences and contour plots clearly demonstrate the superior accuracy of the iKLS element for membrane structural responses, especially considering the low-fidelity discretization (n^e^ = 4) with few sensors used in iFEM analysis of a complex/curved geometry.

As a summary, using iKLS element allows us to improve the accuracy of shape-sensing analysis, even if a very coarse mesh (with a low number of strain sensors) is used for the analysis. This is because the polynomial degree (p, q) of the NURBS basis functions can be elevated without changing the location of knots; hence, the number of elements (i.e., number of sensors) will remain unchanged. This feature of the isogeometric iFEM formulation is the notable technology that is used in this case study to obtain accurate displacements, even with a low-fidelity iKLS discretization.

**Figure 8:**
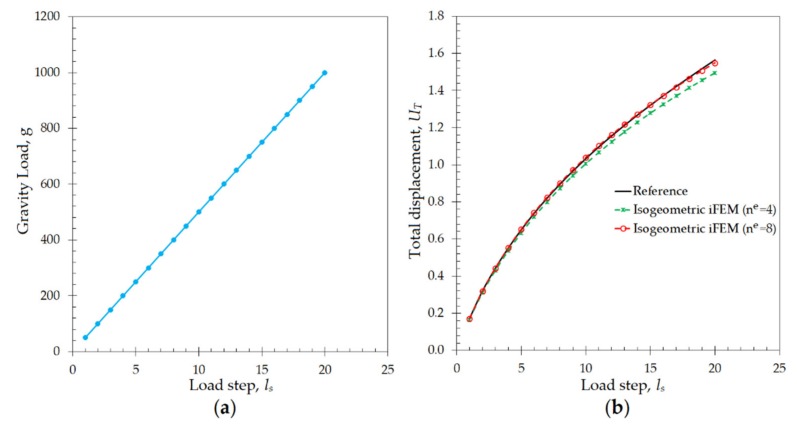
(**a**) Variation of gravity load and (**b**) maximum $U_{T}$ displacements [m] versus increasing number load steps for Scordelis–Lo roof.

Apart from the linear and small deformations, the capability of the isogeometric iFEM formulation for reconstruction of nonlinear/large deformation is also assessed, by performing shape-sensing analyses on the geometry of Scordelis–Lo roof. For this purpose, the gravitational load of the geometry is incrementally increased from 50 to 1000 m/s^2^ for 20 load-steps, $l_{s}$, shown in Figure 8a. Under this loading condition, the direct FEM analysis was performed on the same high-fidelity FEM mesh, by applying the symmetric constraints described previously. This direct structural analysis was conducted in ANSYS Mechanical APDL software, where the option of geometrically nonlinear analysis was turned on to obtain accurate solutions for the large displacements. In this manner, reference large displacement solution, as well as the nonlinear strain data representing the experimental strain measurements, are accurately established. Then, the strain-rosette measurements at the geometric center of each iKLS elements available in the isogeometric discretization are collected from the high-fidelity FEM analysis and transferred to the iFEM analysis for each incremental load-step of $l_{s}=1,2,\ldots ,20$. With this nonlinear strain data variation over the load steps, the isogeometric iFEM analyses are performed for coarse and fine iKLS models, with the element subdivisions of n^e^ = 4 and n^e^ = 8, respectively. 

**Figure 9:**
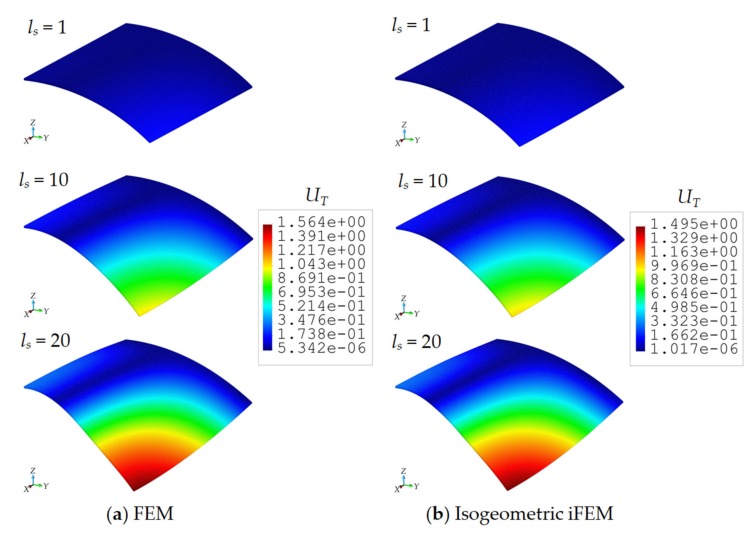
Contour plots of $U_{T}$ displacements on the deformed shape (magnification factor of 3) of Scordelis–Lo roof: Comparison between high-fidelity FEM and isogeometric iFEM (n^e^ = 4) analyses.

As a result of the either iFEM and FEM analyses, the maximum total displacement, $U_{T}=\sqrt{U^{2}+V^{2}+W^{2}}$, is observed at point A located in the roof geometry shown in Figure 5a. The variation of this displacement versus increasing load-steps are compared between isogeometric iFEM (n^e^ = 4 and n^e^ = 8) and reference solutions in Figure 8b. Here, the percent difference between reference analysis and iFEM/iKLS analysis of n^e^ = 4 and n^e^ = 8 for maximum total displacement at load-step of $l_{s}=20$ is approximately 1.04% and 4.40%, respectively. As can be clearly observed from Figure 8b that the total displacement, $U_{T}$, possesses a nonlinear variation against incremental load and this nonlinearity is accurately captured by using the isogeometric iFEM methodology. In addition to maximum displacement comparisons, the total displacement contours obtained from the iFEM/iKLS (n^e^ = 4) and high-fidelity FEM analyses are compared to each other for different loads-steps in Figure 9. For a prudent comparison between reconstructed and reference deformed shapes, these displacement contours are plotted over deformed configurations of the roof geometries. According to the displacement contours, the spatial variations of the total displacement obtained from iFEM are almost identical to those of reference solutions for different load steps. Moreover, in the case of increasing load, the nonlinear deformed shapes produced by iKLS model (n^e^ = 4) conforms accurately to the reference deformed configurations, thereby demonstrating the highly predictive capability of the isogeometric iFEM formulation for nonlinear shape sensing, even with a low number of sensors. Although the present formulation does not accommodate the nonlinear strain components in the analytical strain definitions, the linear analytical strains can still be used to approximate the effect of nonlinear and large deformations, as long as the in situ experimental strains contain the nonlinear strain contributions. This is because the weighted least-squares functional of iFEM aims to match the analytical definition to those of experimental input strains. If the experimental strains include nonlinear effects (i.e., large enough as in the case of large deformations), then a linear analytical strain definition can even be capable of nonlinear deformation reconstruction. Hence, it can be concluded that, in addition to the linear displacement, the present isogeometric iFEM formulation is a viable technology for predicting sufficiently accurate nonlinear displacements with a coarse isogeometric discretization.

### 3.2. Hemisphere

A hemispheric shell subjected to four different concentrated loads (with magnitude of *F* = 2 N) has a radius of *r* = 10 m and a thickness of 2*h* = 40 mm. The prescribed boundary conditions are the minimum required to prevent rigid body motions. In other words, the apex of the hemisphere along *Z*-direction needs to be fixed in order to eliminate the rigid body motion. The hemisphere is made of an isotropic material with an elastic modulus of *E* = 68.25 MPa and a Poisson’s ratio of *v* = 0.3. Morley and Morris [52] originally solved this problem, and after that, Mac Neal and Harder [48] and Belytschko et al. [49] studied this hemisphere problem in detail.

**Figure 10:**
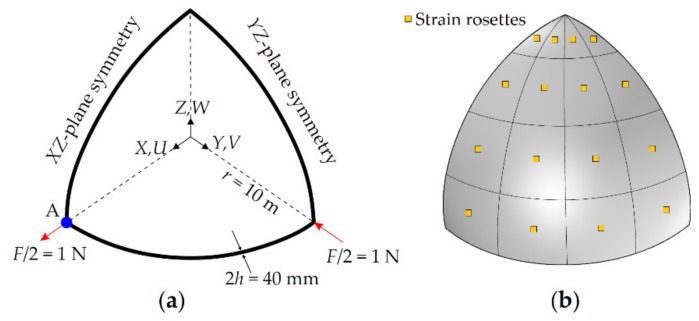
(**a**) Pinched hemisphere with symmetric boundary conditions; (**b**) its discretization (n^e^ = 4), using iKLS elements with top- and bottom-surface strain rosettes per each element [41].

In contrast to the Scordelis–Lo roof problem solved in the previous section, this hemisphere problem is challenging in terms of demonstrating bending capability of the iKLS element, because it exhibits almost none of the membrane strains. Moreover, doubly curved geometry and concentrated loads make this problem highly sensitive to locking phenomena. Therefore, in this section, the pinched hemisphere is analyzed once again, based on the presented isogeometric iFEM formulation. Similar to the Scordelis–Lo roof problem, it is also possible to take the advantage symmetry for this problem. Thus, the following iFEM and direct FEM models are defined over one quarter of the hemisphere and suitable symmetry constraint and loading boundary conditions are applied as depicted in Figure 10a.

First, an accurate reference solution is established through a convergence study that is performed using direct FEM analysis. The most refined mesh consisted of 7500 uniformly distributed rectangular elements, possessing 45,906 DOF. To examine the accuracy of this high-fidelity FEM analysis, the quantity of interest is the displacement along the direction of the loading *F* at point *A* (refer to Figure 10a), which is represented by the symbol $U_{A}$. The value of this displacement is found as $U_{A}=0.0921\;m$ from the high-fidelity FEM analysis, which is in fairly good agreement with the reference solution, $U_{A}=0.0940\;m$, found in [48]. Thus, the FEM deflections and rotations can be securely used to compute the simulated in situ surface strains in the following iFEM analysis.

In the current iFEM analysis, five different iKLS discretizations are generated by uniformly dividing edges of one quarter of the hemisphere into 2, 4, 6, 8, and 10 segments (i.e., number of element subdivisions, n^e^), respectively. Similar to the iKLS discretization used for Scordelis–Lo roof, the polynomial degrees of the NURBS shape functions are fixed to p=q=8, and C^1^-continuity across an interior element boundary is ensured for each iKLS model. Moreover, two strain rosettes are located per each element of each iKLS model, one on the centroid of the top surface and the other one on the centroid of the bottom surface. According to this arrangement of in situ strain rosettes, an example of strain rosette configurations is illustrated for iKLS discretization (n^e^ = 4) in Figure 10b. For a clear assessment of the accuracy of the displacement predictions, the normalized displacements ($\bar{U}$, $\bar{V}$, $\bar{W}$) given by Equation (31) are used herein with the normalization factors, $U_{\max}^{\mathrm{FEM}}=0.0921\;m$, $V_{\max}^{\mathrm{FEM}}=-0.0921\;m$, and $W_{\max}^{\mathrm{FEM}}=0.0457\;m$, that are maximum values of the displacements obtained from the high-fidelity FEM analysis of the hemisphere.

**Figure 11:**
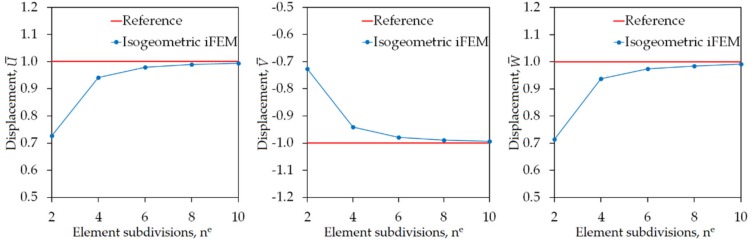
Convergence of maximum values of $\bar{U}$,$\bar{V}$,$\bar{W}$ displacements, versus increasing number of element subdivisions n^e^ for hemisphere.

In Figure 11, the maximum values of displacements ($\bar{U}$, $\bar{V}$, $\bar{W}$) are compared between iFEM and reference FEM analysis for a varying number of element subdivisions (n^e^) of the hemisphere, respectively. These plots demonstrate the following two observations: (1) once the element subdivision becomes n^e^ = 4, the percent differences between iFEM and FEM solutions for all three displacements are approximately 6%, and (2) the convergence rate of the iFEM predictions to reference solutions follows a similar path for all three displacements. These observations confirm the superior bending capability of the iKLS element, even if a low-fidelity discretization (n^e^ = 4) with few number of sensors (i.e., 16×2=32 strain rosettes in total) is used in the shape-sensing analysis. 

**Figure 12:**
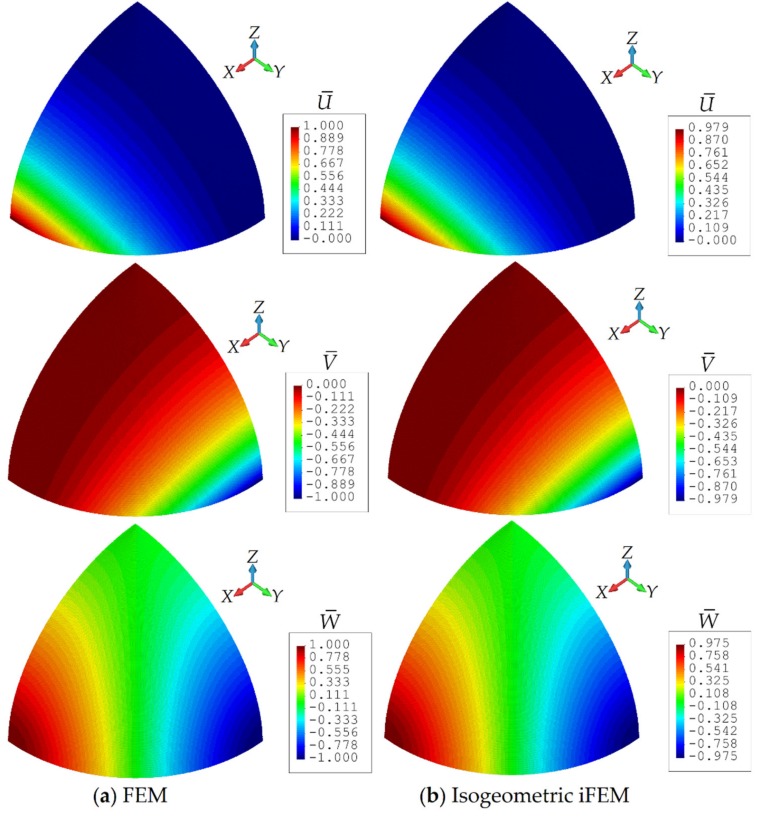
Contour plots of $\bar{U}$,$\bar{V}$,$\bar{W}$ displacements for hemisphere: Comparison between (**a**) high-fidelity FEM and (**b**) isogeometric iFEM (n^e^ = 6) analyses.

In addition, the contour plots for the $\bar{U}$, $\bar{V}$, and $\bar{W}$ displacements are depicted in Figure 12, where contour plots for isogeometric iFEM analysis are graphically almost identical to those of FEM analysis. Note that, in these figures, the displacement results for the iFEM analysis are predicted using the iKLS discretization (n^e^ = 6), with 36×2 strain rosettes in total. As clearly presented in Figure 12, the percent difference between the iFEM and FEM estimates for the maximum values of $\bar{U}$, $\bar{V}$, and $\bar{W}$ are about 2.1%, 2.1%, and 2.5%, respectively. Remarkably, these predictions demonstrate the high quality precision of isogeometric iFEM solutions for the shape-sensing analysis of a complex/curved geometry.

### 3.3. Hyperbolic Paraboloid

**Figure 13:**
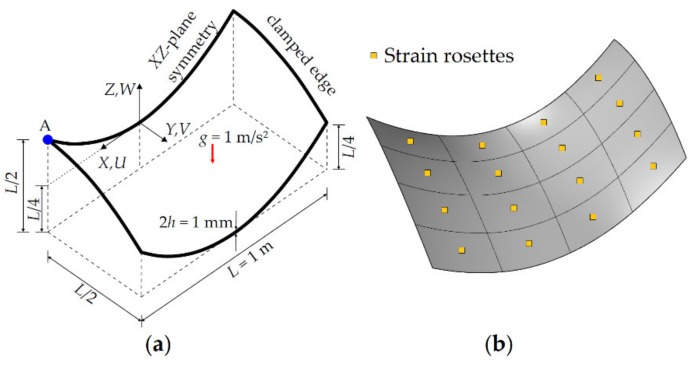
(**a**) Hyperbolic paraboloid with symmetric boundary conditions; (**b**) its discretization (n^e^ = 4) using iKLS elements with top- and bottom-surface strain rosettes per each element.

A partly clamped hyperbolic paraboloid subjected to its self-weight has a length of *L* = 1 m and a uniform thickness of 2*h* = 1 mm. The mid-surface of the hyperbolic paraboloid is defined as $Z=X^{2}-Y^{2}$, where the domain of the surface is defined over (X,Y)∈[−L/2;L/2]. It is worth noting, herein, that this surface can be readily constructed using second order B-splines. The hyperbolic paraboloid is made of an isotropic material having an elastic modulus of *E* = 200 GPa, a Poisson’s ratio of *v* = 0.3, and a density of *ρ* = 8000 kg/m^3^. As presented in Figure 13a, the mid-surface is clamped from the edge located at X=−L/2 and a unit gravitational load of *g* = 1 m/s^2^ is applied to the mid-surface.

This problem was originally solved in Chapelle and Bathe [53], where it was suggested as a good test for locking behavior. Then, Bathe et al. [50] also performed a FEM analysis of this problem and confirmed that it is an excellent test for locking in bending-dominated situations. Therefore, in this section, a shape-sensing analysis of the presented hyperbolic paraboloid is performed based on the isogeometric iFEM methodology, in order to better assess the capability of the iKLS element against the locking phenomenon. The prescribed boundary conditions and geometry are suitable to take advantage of the symmetry plane. Therefore, as shown in Figure 13a, only half of the hyperbolic paraboloid can be modelled while applying the appropriate symmetry boundary conditions. Utilizing an in-house FEM code, an FEM convergence study was carried out to establish an accurate reference solution for this problem. The highest fidelity mesh has 22,500 uniformly distributed rectangular elements and 136,806 DOF. To assess the accuracy of the FEM convergence study, the quantity of reference is denoted by the symbol $W_{A}$ representing the vertical displacement along *Z*-direction at point *A*, i.e., the midpoint of the edge located at X=+L/2 (refer to Figure 13a). The reference solution was found as $W_{A}=-6.3941\;\mathrm{mm}$ in Bathe et al. [50], whereas the high-fidelity FEM analysis predicts this vertical displacement as $W_{A}=-6.4061\;m$, which agrees with its associated reference solution. Hence, the FEM deflections and rotations are directly used to compute the simulated in situ strains.

In the following iFEM analysis, the hyperbolic paraboloid is analyzed using five different iKLS meshes, where edges of the geometry are uniformly divided into the same number of element subdivisions (n^e^ = 2, 4, 6, 8, 10), respectively. Similar to the previous case studies, the polynomial order is defined as p=q=8 for the NURBS shape functions. However, as opposed to the previous case studies, C^2^-continuous NURBS basis functions are attained across an interior element boundary by arranging multiplicity of the corresponding knot value. Therefore, this arrangement allows the exact paraboloid surface to be encapsulated in the iKLS model. Two strain rosettes are located in each iKLS element at the following positions: (1) on the centroid of the top surface, and (2) on the centroid of the bottom surface. To give an example of strain rosette configurations, the iKLS models (n^e^ = 4) are presented in Figure 13b.

**Figure 14:**
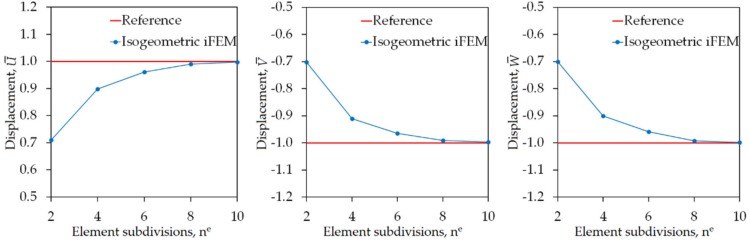
Convergence of maximum values of $\bar{U}$,$\bar{V}$,$\bar{W}$ displacements, versus increasing number of element subdivisions n^e^ for hyperbolic paraboloid.

**Figure 15:**
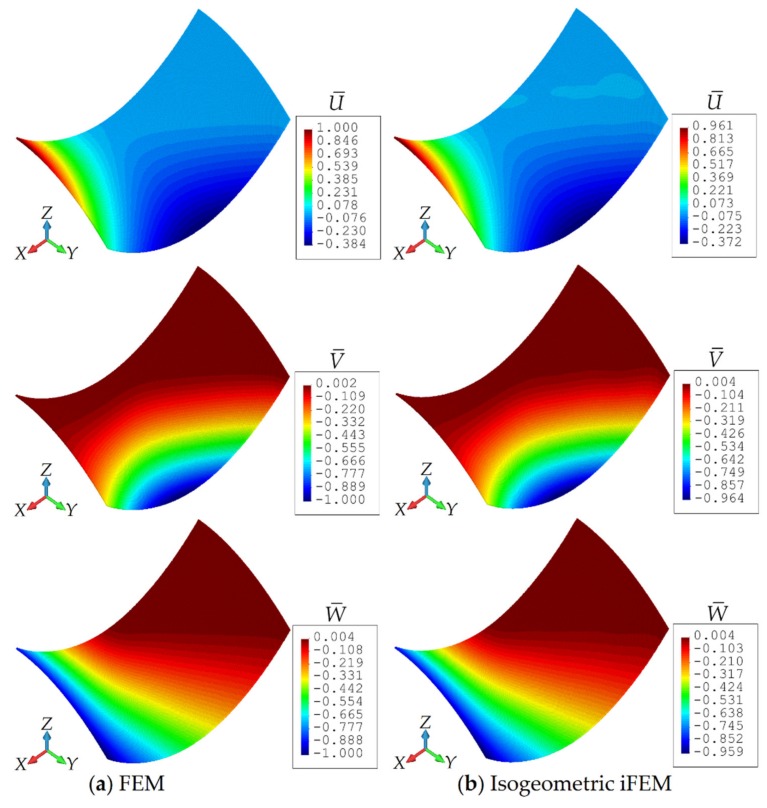
Contour plots of $\bar{U}$,$\bar{V}$,$\bar{W}$ displacements for hyperbolic paraboloid: Comparison between (**a**) high-fidelity FEM and (**b**) isogeometric iFEM (n^e^ = 6) analyses.

Similar to the previous case studies, the normalized displacements ($\bar{U}$, $\bar{V}$, $\bar{W}$) given by Equation (31) are also calculated for the hyperbolic paraboloid in order to clearly examine the precision of the displacement estimates. The following maximum values of the displacements are obtained in the high-fidelity FEM analysis of the hyperbolic paraboloid: $U_{\max}^{\mathrm{FEM}}=3.612\;\mathrm{mm}$, $V_{\max}^{\mathrm{FEM}}=1.927\;\mathrm{mm}$, and $W_{\max}^{\mathrm{FEM}}=7.372\;\mathrm{mm}$, which are used as the normalization factors in Equation (31). As plotted in Figure 14, the maximum values of $\bar{U}$, $\bar{V}$, and $\bar{W}$ are compared between iFEM and reference FEM analysis for varying number of element subdivisions (n^e^), respectively. These results demonstrated that the iFEM predictions for the $\bar{U}$, $\bar{V}$, and $\bar{W}$ displacements convergence to their reference solutions by following a similar pathway. In addition, as can be seen from these graphs, the percentage differences between iFEM and FEM estimates for all three displacements are approximately 30% when iKLS discretization (n^e^ = 2) is used, whereas these percent differences are dramatically reduced to 10% for the case (n^e^ = 4). Finally, iKLS discretization (n^e^ = 10) predicts displacements that are as perfectly accurate as those of the reference solutions. Besides, in Figure 15, contour plots of the $\bar{U}$, $\bar{V}$, and $\bar{W}$ displacements are compared between the isogeometric iFEM and high-fidelity FEM analyses.

In these figures, the iFEM contours correspond to the analysis that uses the iKLS model (n^e^ = 6), with 36×2 strain rosettes in total. The percentage difference between the iFEM and FEM for the maximum values of the $\bar{U}$ displacement is only 3.9%. Similar accuracy is evidenced for the maximum values of others’ displacement, with a percentage difference of 3.6% for the $\bar{V}$ displacement, and 4.1% for the $\bar{W}$ displacement. Both the isogeometric iFEM and direct FEM contours are graphically indistinguishable in the figures. These results demonstrate the superior bending predictions of iKLS element, especially considering the low-fidelity mesh used in the iFEM analysis.

## 4. Conclusions 

This study presents an isogeometric iFEM methodology, which couples the IGA with the iFEM, for shape-sensing analyses of complex (curved) thin plate and shell structures that are instrumented with a network of distributed sensors. In addition, an isogeometric Kirchhoff–Love inverse-shell element (iKLS) is developed for the numerical implementation of the proposed algorithm. The membrane and bending capability of the iKLS element was demonstrated by carrying out several numerical simulations, including Scordelis–Lo roof, pinched hemisphere, and partly clamped hyperbolic paraboloid. In the analysis of these problems, experimentally measured strains are represented by strain results obtained from a high-fidelity solution, using an in-house finite element code. Several types of discretization strategies are examined and comparisons of iFEM and direct FEM displacement solutions are provided. As a result, the membrane robustness and the bending efficiency of the iKLS element has been justified even using the low-fidelity discretization with few strain sensors. The effects of sensor locations, number of sensors, and the iFEM discretization of the geometry on solution accuracy are pondered. It has been demonstrated that the iKLS element has the advantage of simply modelling the curved shell structures because of its NURBS-based nature. Moreover, it has been confirmed that even if a very coarse mesh (with a low number of strain sensors) is used in the shape-sensing analysis, the isogeometric iFEM approach provides superior linear/nonlinear displacement solutions. This is because the polynomial degree of the NURBS basis function can be increased without changing the location of knots. Overall, the present results reveal that the isogeometric iFEM methodology is an attractive candidate for the shape sensing and real-time monitoring of shell structures undergoing small/large displacements. 

## Appendix A

The matrix of shape function derivatives that are used to establish strain-displacement relation, as given in Equation (25a), can be explicitly expressed as:

(A1) $$B_{\alpha }=\left[\begin{array}{cccc} B_{\alpha }^{1} & B_{\alpha }^{2} & \ldots & B_{\alpha }^{n_{cp}} \end{array}\right]\;(\alpha =m,b)$$

with

(A2) $$B_{\alpha }^{j}=\left[\begin{array}{ccc} \alpha_{11}^{1j} & \alpha_{11}^{2j} & \alpha_{11}^{3j}\\ \alpha_{22}^{1j} & \alpha_{22}^{2j} & \alpha_{22}^{3j}\\ 2\;\alpha_{12}^{1j} & 2\;\alpha_{12}^{2j} & 2\;\alpha_{12}^{3j} \end{array}\right]\;\;\;\;(j=1,2,\ldots ,n_{cp},\;\;\alpha =m,b)$$

In Equation (A2), the terms related to membrane strain measures and bending curvatures can be defined as:

(A3) $$m_{\alpha \beta }^{ij}=\frac{1}{2}\left[(A_{\beta }\cdot {\hat{e}}_{i})R_{j,\alpha }+(A_{\alpha }\cdot {\hat{e}}_{i})R_{j,\beta }\right]$$

and

(A4) $$\begin{array}{l} b_{\alpha \beta }^{ij}=\frac{1}{2}\left[(A_{3,\beta }\cdot {\hat{e}}_{i})R_{j,\alpha }+(A_{3,\alpha }\cdot {\hat{e}}_{i})R_{j,\beta }\right]\\ \;+\frac{1}{2}\left[(A_{3}\times A_{\beta })\cdot \Theta_{\alpha }^{ij}+(A_{3}\times A_{\alpha })\cdot \Theta_{\beta }^{ij}\right] \end{array}$$

where the term $\Theta_{\alpha }^{ij}$ contains second-order derivatives of the NURBS basis functions and can be explicitly defined as:

(A5) $$\begin{array}{l} \Theta_{\alpha }^{ij}=(A_{3}\cdot {\hat{e}}_{i})\;(\chi_{1}\;R_{j,\alpha 2}-\chi_{2}\;R_{j,1\alpha })\\ \;+(A_{3}\cdot {\hat{e}}_{i})\;(\chi_{1,\alpha }\;R_{j,2}-\chi_{2,\alpha }\;R_{j,1})\\ \;+(A_{3,\alpha }\cdot {\hat{e}}_{i})\;(\chi_{1}\;R_{j,2}-\chi_{2}\;R_{j,1}) \end{array}$$

with

(A6) $$R_{i,11}\equiv R_{i,\xi \xi },\;R_{i,22}\equiv R_{i,\eta \eta },\;R_{i,12}\equiv R_{i,\xi \eta }$$

(A7) $$\chi_{\alpha }=\frac{A_{\alpha }}{\left\|A_{1}\times A_{2}\right\|},\;\chi_{\alpha ,\alpha }=\frac{A_{\alpha ,\alpha }-\chi_{\alpha }\left[A_{1,\alpha }\cdot (A_{2}\times A_{3})+A_{2,\alpha }\cdot (A_{3}\times A_{1})\right]}{\left\|A_{1}\times A_{2}\right\|}$$

and

(A8) $$A_{3,\alpha }=\frac{A_{1,\alpha }\times A_{2}+A_{1}\times A_{2,\alpha }-A_{3}\left[A_{1,\alpha }\cdot (A_{2}\times A_{3})+A_{2,\alpha }\cdot (A_{3}\times A_{1})\right]}{\left\|A_{1}\times A_{2}\right\|}$$

where the subscripts/superscripts vary as i=1,2,3, $j=1,2,\ldots ,n_{cp}$, α=1,2, and β=1,2.
